# Creatine transporter deficiency impairs stress adaptation and brain energetics homeostasis

**DOI:** 10.1172/jci.insight.140173

**Published:** 2021-09-08

**Authors:** Hong-Ru Chen, Xiaohui Zhang-Brotzge, Yury M. Morozov, Yuancheng Li, Siming Wang, Helen Heju Zhang, Irena S. Kuan, Elizabeth M. Fugate, Hui Mao, Yu-Yo Sun, Pasko Rakic, Diana M. Lindquist, Ton DeGrauw, Chia-Yi Kuan

**Affiliations:** 1 Department of Neurosciences, University of Virginia School of Medicine, Charlottesville, Virginia, USA.; 2 Department of Pediatrics, Division of Neurology, Emory University, Atlanta, Georgia, USA.; 3 Department of Neuroscience, Yale University School of Medicine, New Haven, Connecticut, USA.; 4 Department of Radiology and Imaging Sciences, Emory University, Atlanta, Georgia, USA.; 5 Department of Chemistry, Georgia State University, Atlanta, Georgia, USA.; 6 Gene Edit Biolab, Atlanta, Georgia, USA.; 7 Imaging Research Center, Department of Radiology, Cincinnati Children’s Hospital Medical Center, Cincinnati, Ohio, USA.

**Keywords:** Metabolism, Neuroscience, Autophagy, Bioenergetics, Neurological disorders

## Abstract

The creatine transporter (CrT) maintains brain creatine (Cr) levels, but the effects of its deficiency on energetics adaptation under stress remain unclear. There are also no effective treatments for CrT deficiency, the second most common cause of X-linked intellectual disabilities. Herein, we examined the consequences of CrT deficiency in brain energetics and stress-adaptation responses plus the effects of intranasal Cr supplementation. We found that CrT-deficient (CrT^–/y^) mice harbored dendritic spine and synaptic dysgenesis. Nurtured newborn CrT^–/y^ mice maintained baseline brain ATP levels, with a trend toward signaling imbalance between the p-AMPK/autophagy and mTOR pathways. Starvation elevated the signaling imbalance and reduced brain ATP levels in P3 CrT^–/y^ mice. Similarly, CrT^–/y^ neurons and P10 CrT^–/y^ mice showed an imbalance between autophagy and mTOR signaling pathways and greater susceptibility to cerebral hypoxia-ischemia and ischemic insults. Notably, intranasal administration of Cr after cerebral ischemia increased the brain Cr/*N*-acetylaspartate ratio, partially averted the signaling imbalance, and reduced infarct size more potently than intraperitoneal Cr injection. These findings suggest important functions for CrT and Cr in preserving the homeostasis of brain energetics in stress conditions. Moreover, intranasal Cr supplementation may be an effective treatment for congenital CrT deficiency and acute brain injury.

## Introduction

Creatine (Cr) and phosphocreatine (PCr) show the highest concentration in tissues that require constant or rapid energy supply, including skeletal muscles, heart, and brain (1, 2). Cr/PCr has energy-shuttling functions in skeletal muscles plus neuroprotective and cognition-enhancing effects in the brain (3–5). For example, the addition of Cr delays the onset of anoxia-induced obliteration of the electrical activity in brain slices (6). Oral Cr supplementation improves the cognitive performance of healthy adults in a hypoxic or normoxic condition (7, 8). Conversely, reductions in Cr/PCr levels and Cr kinase activity have been observed in neurodegenerative diseases (4, 9). Children with cerebral Cr deficiency syndromes also suffer from intellectual disabilities, autism, or seizures (10, 11). However, the neuroprotective potential of Cr/PCr is yet to be harnessed for treating acute brain injury, mainly due to a sluggish blood-to-brain transport of Cr that is mediated by the Cr transporter (CrT) (12, 13).

Our daily supply of Cr primarily comes from de novo Cr biosynthesis and is supplemented by food (1, 2). De novo Cr biosynthesis begins with the formation of guanidinoacetate (GAA) from arginine and glycine that is mediated by arginine/glycine amidinotransferase (AGAT) in the kidney. GAA is then methylated by guanidinoacetate methyltransferase (GAMT) in the liver to become Cr, which enters the blood circulation (1, 2). The uptake of Cr against a concentration gradient in the brain is assisted by specific transporters at the blood-brain-barrier (BBB) (1, 2). Although there is evidence of de novo Cr synthesis in rodent embryonic brains, whether these reactions exist in adult human brains is uncertain (14).

In infants with AGAT or GAMT deficiency, two rare autosomal recessive diseases, the brain and plasma Cr levels are severely reduced, but recover to the normal range after oral Cr supplementation (10, 11). In contrast, children with CrT deficiency (the second most common cause of X-linked intellectual disabilities) show near absence of brain Cr peaks on magnetic resonance microscopy (MRS) despite normal plasma Cr concentrations, a pattern that suggests defects in blood-to-brain transport of Cr (10, 15). Indeed, CrT-deficient children harbor mutations in the *SLC6A8* gene that belongs to the solute carrier 6 (SLC6) family and show poor responses to oral Cr supplementation (11, 16, 17). These clinical features suggest a critical role for CrT in transporting Cr across the BBB, which requires approximately 2 weeks to restore brain Cr levels in GAMT-null mice by daily supplement of 2 g/kg Cr (13, 18). The slow kinetics of Cr transport across the BBB may explain why prophylactic, but not post-stroke intraperitoneal (i.p.) injection, of Cr reduced the infarct size in experimental stroke (12). Hence, it is important to develop more effective Cr-supplementation methods to harness the neuroprotective potential of Cr.

Five lines of transgenic CrT-deficient mice have been generated to date (19–23). These CrT-mutant mice all show cognition impairment and reduced brain Cr concentrations, but their responses to stress stimuli are yet to be determined. To this end, we generated a line of CrT-null (CrT^–/y^) mice using the knockout-first strategy (24). These CrT-null mice showed dendritic spine and synaptic dysgenesis, signaling imbalance between the p-AMPK/autophagy and mTOR pathways, and increased susceptibility to multiple brain insults. Notably, post-stroke intranasal (i.n.) application of Cr partially averted the signaling imbalance and reduced brain infarction in both wild-type and CrT-null mice. These results provide insight into the functions of Cr and CrT in maintaining brain energetics homeostasis and stress adaptation. They also suggest a new approach for Cr supplementation that may be used to treat CrT deficiency and acute brain injury.

## Results

### CrT^–/y^ mice show reduced brain Cr levels plus dendritic spine and synapse dysgenesis.

**Figure 1:**
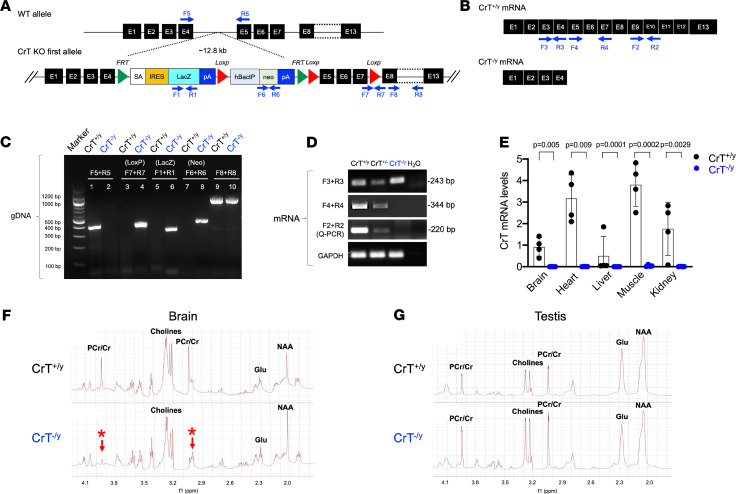
Generation of CrT-null mice. (**A**) The scheme to generate CrT-null (CrT^–/y^) mice using the knockout-first strategy via an ES cell line from the NIH KOMP Repository (CSD24513). The locations of PCR primers to detect the CrT-targeted genomic allele are indicated. (**B**) Schematic of primer design for RT-qPCR analysis of different regions of the CrT (*Slc6a8*) mRNA. (**C**) PCR analysis of the genomic DNA of CrT^+/y^ and CrT^–/y^ mice to verify the wild-type and knockout alleles. The RT-PCR product (243 bp) from primers F3 and R3 corresponds to the region overlapping exon 3 and exon 4 sequences of both CrT^+/y^ and CrT^–/y^ cDNA; the 344 bp and 220 bp RT-qPCR products from primers F4 and R4 and F2 and R2, respectively, correspond to the region overlapping exon 5 to exon 10, which was missing in the CrT^–/y^ cDNA. (**D**) PCR analysis of different regions of the CrT mRNA of CrT^+/y^ and CrT^–/y^ mice. (**E**) RT-qPCR showed the absence of full-length CrT mRNAs in the brain, heart, liver, skeletal muscles, and kidney in CrT^–/y^ mice (*n* = 4). (**F** and **G**) Proton–HR-MAS NMR showed severe reduction in Cr/PCr peaks in the brain (arrows and asterisks), but not the testis, of CrT^–/y^ mice (*n* = 5 for each genotype). All data are shown as mean ± SEM. All *P* values were determined by Student’s *t* test.

We used CrT-targeted (*Slc6a8*-targeted) ES cells in the NIH KOMP Repository [tm1a(KOMP)Wtsi] to generate heterozygous CrT–knockout-first (CrT^+/–^) mutants that contain the IRES:*lacZ* trapping cassette (24) and backcrossed them with wild-type (CrT^+/y^) C57BL/6J mice for more than 6 generations. The CrT–knockout-first allele in CrT-null (CrT^–/y^) mice was confirmed by PCR (Figure 1, A and C). Reverse transcription quantitative PCR (RT-qPCR) analysis validated the absence of full-length CrT (*Slc6a8*) mRNA in the brain, heart, liver, kidney, and skeletal muscles in 5-month-old CrT^–/y^ mice (Figure 1, B, D, and E; *n* = 4 for each genotype). Immunoblotting showed restricted expression of AGAT and GAMT in the kidney and liver, respectively, in wild-type and CrT^–/y^ adult mice (*n* = 4 for each genotype) (Supplemental Figure 1A; supplemental material available online with this article; https://doi.org/10.1172/jci.insight.140173DS1). Proton–high-resolution magic angle spinning (proton–HR-MAS) NMR showed marked reduction of the Cr/PCr peaks in 5-month-old CrT^–/y^ mouse brains when compared with CrT^+/y^ mouse brains (Figure 1F; *n* > 5 for each genotype). CrT^–/y^ mice also showed diminished Cr/PCr peaks in the heart and skeletal muscles, but not in the testes, similar to previous lines of CrT-mutant mice (19, 22) (Supplemental Figure 1, B and C, and Figure 1G; *n* > 5 for each genotype). These results confirmed the Cr deficiency phenotype in CrT^–/y^ mice.

**Figure 2:**
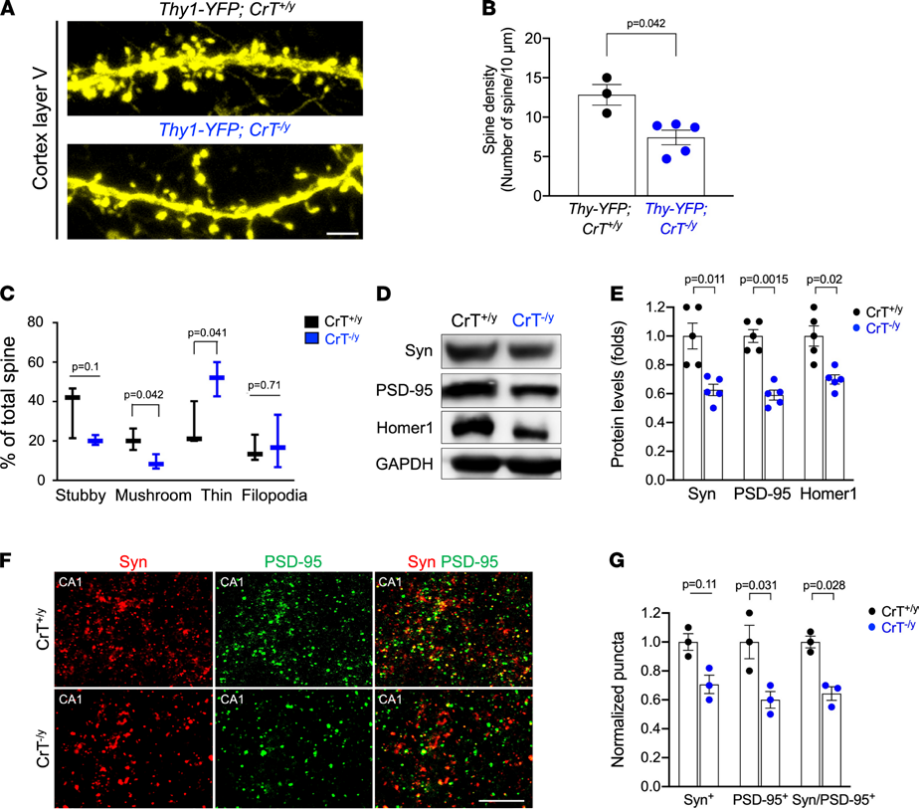
Dendritic spine dysgenesis and synaptic reduction in CrT^–/y^ mice. (**A** and **B**) CrT^+/–^ and Thy1-YFP mice were crossed to assist visualization of dendritic spines in the cortical layer V neurons in CrT^+/y^ versus CrT^–/y^ mice. (**A**) Shown are typical images of dendritic spines in 3-month-old Thy1-YFP; CrT^+/y^ and Thy1-YFP; CrT^–/y^ mice. Scale bar: 5 μm. (**B**) Thy1-YFP; CrT^–/y^ mice (*n* = 5) showed significantly lower spinal density than Thy1-YFP; CrT^+/y^ mice (*n* = 3). (**C**) Spine classification by Imaris software revealed a reduction in the mature, mushroom subtype and an increase in the immature, thin subtype in Thy1-YFP; CrT^–/y^ neurons. (**D** and **E**) Immunoblotting showed significant reduction in postsynaptic proteins (PSD-95 and Homer1), but not presynaptic synaptotagmin (Syn) in the hippocampus of 3-month-old CrT^–/y^ mice compared with CrT^+/y^ mice (*n* = 5 for each genotype). (**F** and **G**) Confocal laser microscopy showed reduced synaptic densities (colocalized anti-Syn and anti–PSD-95 puncta) in CA1 hippocampal neurons in CrT^–/y^ mice compared with CrT^+/y^ mice (*n* = 3 for each genotype). Scale bar: 50 μm. All data are shown as mean ± SEM. All *P* values were determined by Student’s *t* test.

Next, we crossed CrT^+/–^ mice with Thy1-YFP mice to examine the effects of cerebral Cr deficiency on dendritic spine morphogenesis (Figure 2A). We found a significant reduction in spine density, especially the mature, mushroom subtype, and an increase in the immature thin subtype in layer V cortical neurons in Thy1-YFP; CrT^–/y^ mice (Figure 2, B and C; *n* = 3 for the CrT^+/y^ and *n* = 5 for CrT^–/y^ genotype). To evaluate the effects of Cr deficiency on synaptogenesis, we used immunoblotting to compare the hippocampus of 3-month-old CrT^+/y^ and CrT^–/y^ mice, and found a significant reduction in postsynaptic PSD-95, Homer1, and presynaptic synaptotagmin proteins (Figure 2, D and E; *n* = 5 for each genotype). Likewise, quantification of the anti-synaptotagmin, anti–PSD-95, and colocalized synaptotagmin/PSD-95 puncta suggested a significant reduction in synapses in CA1 hippocampal neurons in CrT^–/y^ mice, primarily driven by diminished anti–PSD-95 immunopuncta (Figure 2, F and G; *n* = 3 for each genotype). These results suggest that Cr/CrT deficiency may lead to dendritic spine and synapse dysgenesis that has been implicated in intellectual disabilities (25).

### CrT^–/y^ neonates have greater sensitivity to starvation-induced autophagy in the brain.

**Figure 3:**
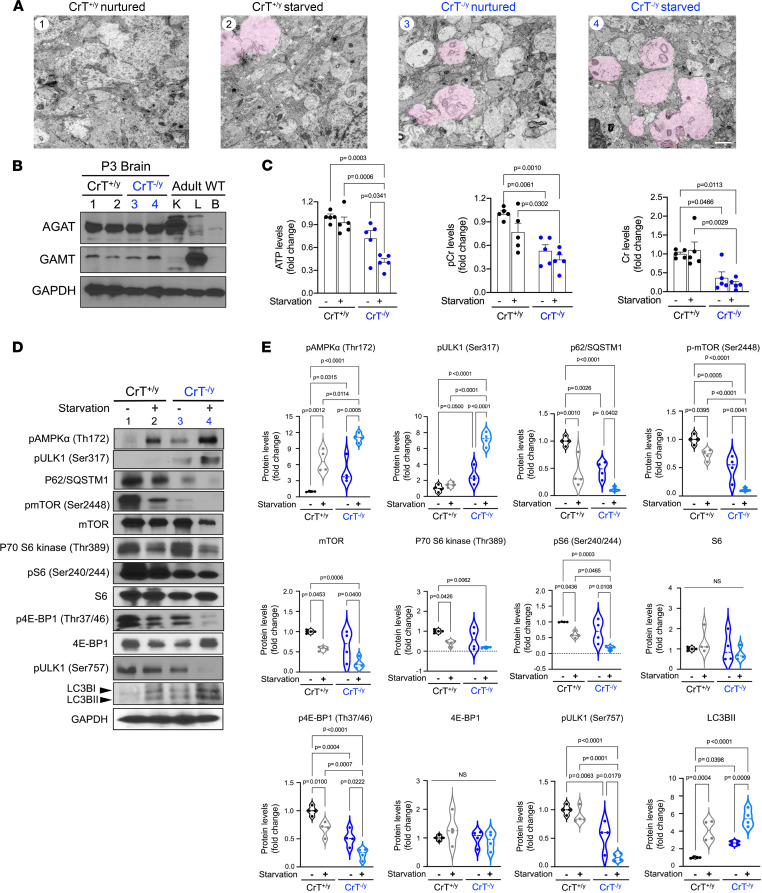
Imbalance between p-AMPK/autophagy and mTOR signaling in CrT^–/y^ neonates. (**A**) EM showed more membrane whorls (arrows) and large lucid cytoplasm (pink-colored) in the neocortical neuropil of 3-day-old CrT^–/y^ mice, particularly after 12 hours of starvation/separation from the dam (*n* = 3 for each condition). The numbering 1–4 for the indicated condition is used in all panels of this figure. Scale bar: 1 μm. (**B**) Immunoblot detection of AGAT and GAMT in P3 CrT^+/y^ and CrT^–/y^ mice (*n* > 3 for each). Note the expression of AGAT and GAMT in P3 mouse brains with or without starvation, but not in the adult brain. (**C**) LC-MS measurement of the brain ATP, Cr, and PCr levels in P3 CrT^+/y^ and CrT^–/y^ neonates with or without starvation (*n* = 5 for each group). (**D** and **E**) Immunoblotting analysis and quantification of the p-AMPK/autophagy and mTOR signaling pathways in P3 CrT^+/y^ and CrT^-/y^ mouse brains, with or without starvation as indicated (*n* = 4 for each). The violin plots in **E** are representative of 3 independent experiments, with *n* = 4 biological replicates; all other data are shown as mean ± SEM. All statistical analyses were performed using 2-way ANOVA followed by Tukey’s multiple comparison post hoc test.

Next, we assessed the effects of CrT deficiency on brain energetics and stress adaptation in 4 experimental models. The first model that we examined is neonatal starvation in 3-day-old pups, a well-known stimulus for autophagy induction without cell death in the brain (26). We applied electron microscopy (EM), liquid chromatography–mass spectrometry (LC-MS), and immunoblotting to compare the brains of P3 CrT^+/y^ versus CrT^–/y^ mice, with or without 12 hours of separation from the dam. The experimental conditions were labeled as: 1, nurtured CrT^+/y^ neonates; 2, starved CrT^+/y^ neonates; 3, nurtured CrT^–/y^ neonates; and 4, starved CrT^–/y^ neonates, throughout Figure 3 for comparison of the findings in different assays.

The EM analysis showed an increase in membrane whorls and empty cytoplasm (arrows and pink-colored area in Figure 3A) in starved CrT^+/y^ and nurtured CrT^–/y^ neonatal brains, and these indicators of autophagy were more intensified in the starved CrT^–/y^ neonatal brains (Figure 3A; *n* = 3 for each group). Interestingly, 3-day-old CrT^+/y^ and CrT^–/y^ mouse brains contained AGAT, and to a lesser degree GAMT expression, in contrast to adult brains, both with and without starvation (Figure 3B; *n* = 3 for each group). LC-MS showed that nurtured P3 CrT^–/y^ mice have a near-normal ATP level in the brain, despite a larger reduction in PCr and Cr (Figure 3C; *n* = 5 for each condition). However, their brain ATP levels were markedly reduced after starvation, in contrast to starved CrT^+/y^ mice (Figure 3C).

Immunoblotting showed an increase in p-AMPKα (Thr172), p-ULK1 (Ser317), LC3B-II, and a reduction in p62/SQSTM1 and p-ULK1 (Ser757) — all signs of autophagy induction (27, 28) — in nurtured CrT^–/y^ neonatal brains (condition 3) and to a lesser degree in starved CrT^+/y^ neonatal brains (condition 2). These changes were more pronounced in CrT^–/y^ neonates after 12 hours of starvation (condition 4) (Figure 3, D and E; *n* = 4 for each condition). Conversely, the brains of starved CrT^+/y^ (condition 2) and nurtured CrT^–/y^ mice (condition 3) showed signs of suppressed mTOR signaling, including reduced p-mTOR (Ser2448) and p-4E-BP1 (Thr37/46). The starved CrT^–/y^ mouse brains also showed these aberrations plus additional signs of mTOR signaling repression, including a reduction in mTOR and p-S6 (Ser240/244) (Figure 3, D and E).

These results suggested that CrT^–/y^ neonates utilized an imbalance between the autophagy (catabolism) and mTOR (anabolism) signaling activity to maintain the brain energetics baseline. This signaling imbalance was amplified without causing obvious brain damage after starvation in CrT^–/y^ neonates, likely due to modest energetics stress in this model.

### CrT deficiency produces greater mitochondrial ROS and reduced viability after in vivo and in vitro hypoxia-ischemia.

**Figure 4:**
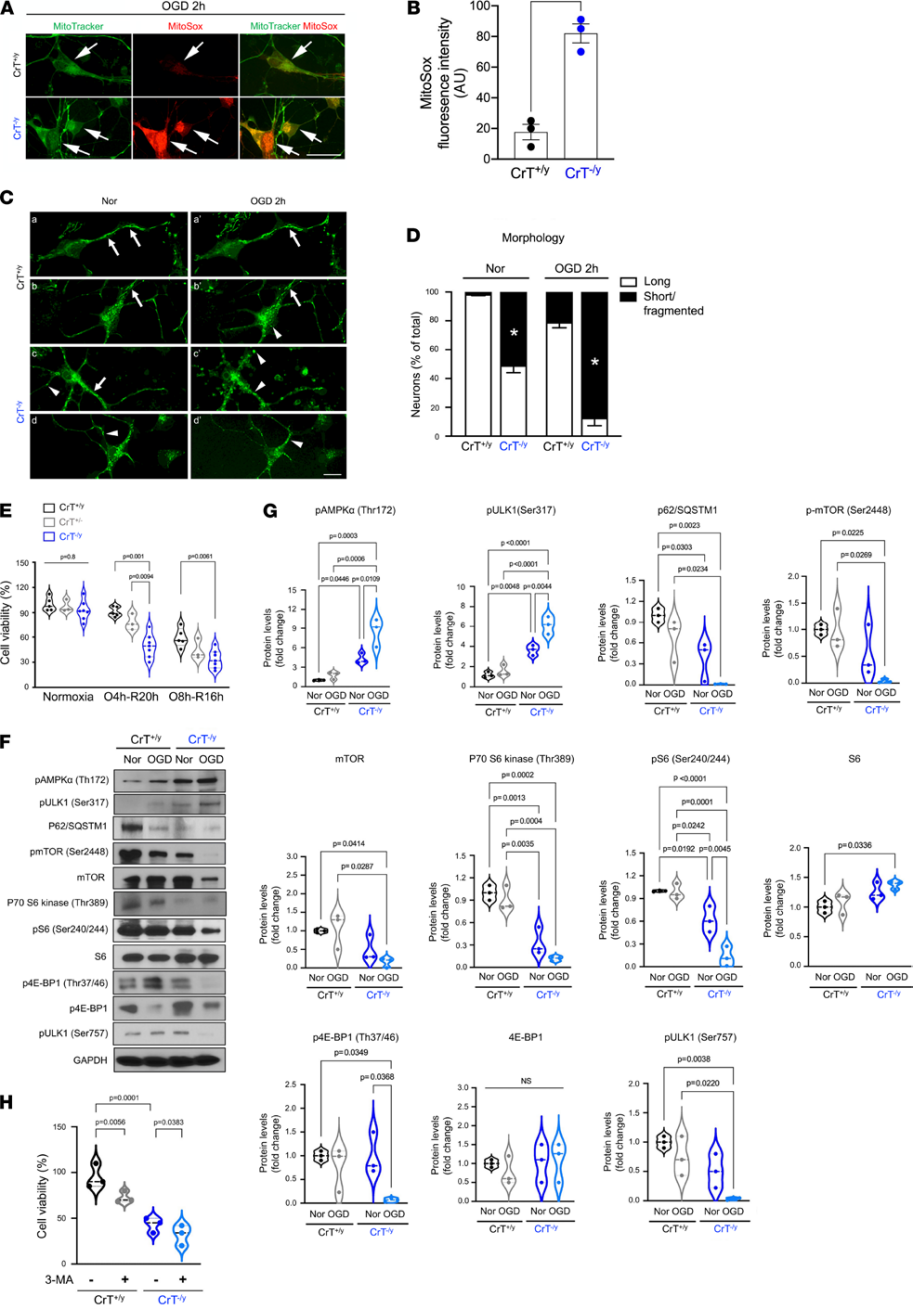
CrT deficiency increases mitochondrial ROS and reduces neuronal viability after oxygen-glucose deprivation. (**A** and **B**) CrT^+/y^ and CrT^–/y^ cortical neurons were stained with MitoTracker and 4 μM MitoSox Red and visualized by fluorescence microscopy after challenge with 2 hours of oxygen-glucose deprivation (OGD). Mitochondrial ROS production was quantified by measurement of MitoSOX fluorescence intensity. The mitochondrial subcellular location of MitoSOX was visualized by colabeling with MitoTracker Green using a Leica SP8 confocal microscope. *n* = 3 sets of cultures. Scale bar: 10 μm. (**C** and **D**) Control CrT^+/y^ cortical neurons exhibited normal long/tubular mitochondrial morphology, whereas those exposed to 2 hours of OGD showed increased short/mitochondrial fragmentation in a time-dependent manner. CrT^–/y^ cortical neurons showed increased short/mitochondrial fragmentation both in normoxia (Nor) and after OGD compared with CrT^+/y^ neurons. The mitochondrial morphology was visualized by MitoTracker Green. Note the presence of long (arrows) and short mitochondria (arrowheads). Scale bar: 10 μm. (**E**) CrT^–/y^ cortical neurons showed reduced viability after 4- or 8-hour OGD, followed by a 20- or 16-hour recovery, respectively, compared with CrT^+/y^ neurons (*n* = 3–7 sets of cultures). (**F** and **G**) Immunoblot analysis and quantification of the p-AMPK/autophagy and mTOR signaling pathway activity in CrT^+/y^ and CrT^–/y^ cortical neurons in normoxia (Nor) or 4 hours after OGD (*n* = 3 for each condition). (**H**) Influence of autophagy inhibitor 3-MA on cell viability of OGD-challenged CrT^+/y^ and CrT^–/y^ cortical neurons. Violin plots in **E**, **G**, and **H** are representative of 3 independent experiments, with *n* = 3–7 (**E**) or 3 (**G** and **H**) biological replicates; all other data are shown as mean ± SEM. Statistical significance was determined using Student’s *t* test (**B** and **D**), 1-way ANOVA followed by Tukey’s multiple comparisons post hoc test (**E**), or 2-way ANOVA followed by Tukey’s multiple comparisons post hoc test (**G** and **H**).

Next, we tested whether CrT deficiency impairs mitochondrial functions and neuronal viability after in vitro oxygen-glucose deprivation (OGD), or in vivo neonatal hypoxia-ischemia (HI), or both. CrT^+/y^ and CrT^–/y^ cortical neurons at 7 days in vitro (DIV) were stained with MitoSox Red and MitoTracker dyes after 2 hours of OGD (Figure 4, A–D). CrT^–/y^ cortical neurons show increased MitoSox Red fluorescence (an indicator of superoxide), more short, fragmented mitochondria (stained by MitoTracker), and abnormal mitochondrial distribution compared with CrT^+/y^ neurons (Figure 4, A–D, and Supplemental Figure 2). CrT^–/y^ neurons also showed reduced survival after 4 hours or 8 hours of OGD, followed by 20 (“O4h-R20h” in the figure) or 16 hours of reoxygenation (“O8h-R16h” in the figure), respectively, compared with CrT^+/y^ neurons (Figure 4E; *n* = 3–7 sets of cultures as indicated). Similar to the pattern in neonatal starvation, the immunoblotting analysis showed an increase in p-AMPKα (Thr172), p-ULK1 (Ser317), and a reduction in p62/SQSTM1, p-ULK1 (Ser757), p-mTOR (Ser2448), p-70 S6 kinase (Thr389), and p-4E-BP1 (Thr37/46) in CrT^–/y^ neurons after OGD, compared with OGD-injured CrT^+/y^ neurons (Figure 4, F and G; *n* = 3 for each condition). Furthermore, application of the autophagy inhibitor 3-methyladenine (3-MA) reduced the viability of both CrT^+/y^ and CrT^–/y^ neurons after OGD (Figure 4H; *n* = 3 for each condition), suggesting that the post-OGD increase in autophagy is a means to maintain neuronal viability under stress.

**Figure 5:**
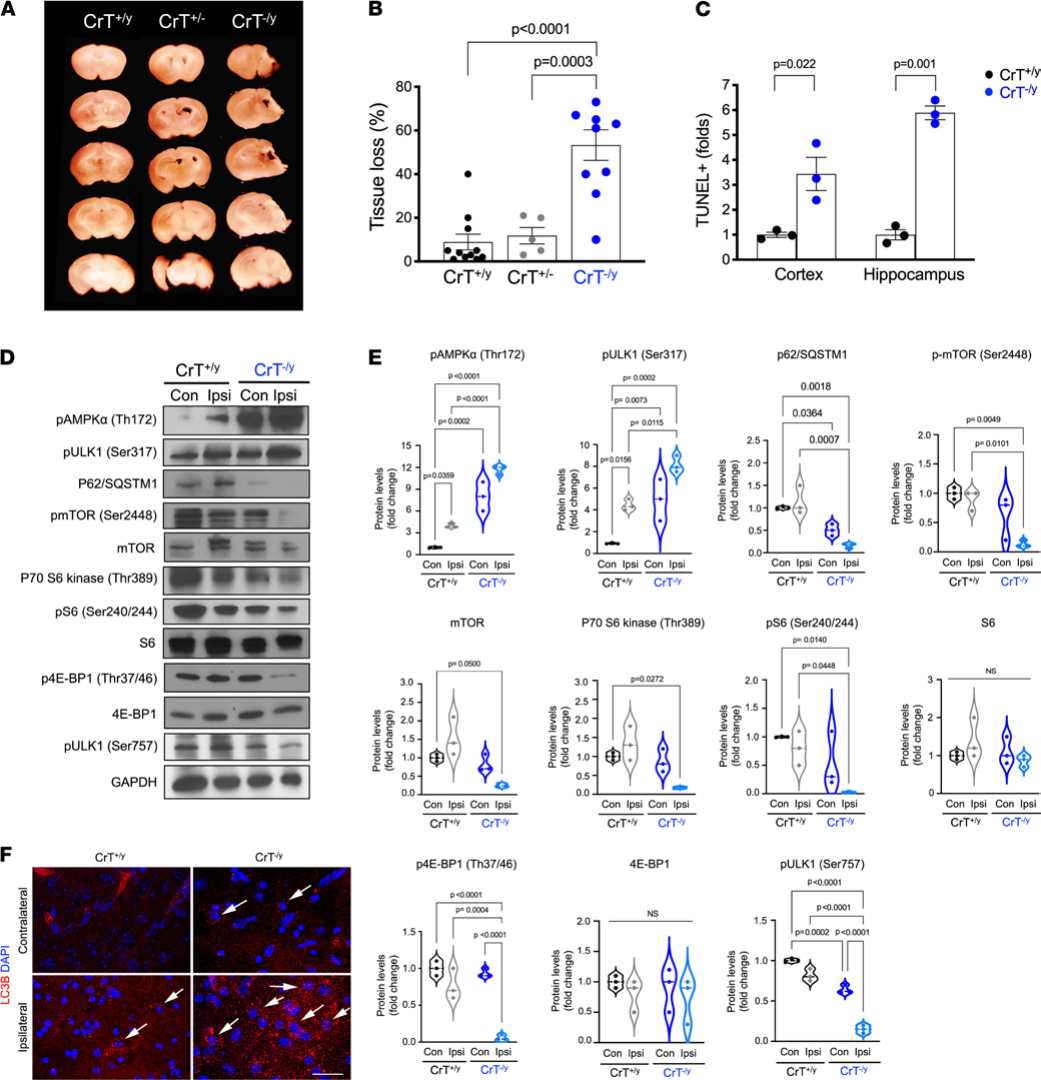
CrT deficiency increases brain damage after neonatal hypoxic-ischemic (HI) insult. (**A** and **B**) Representative brain sections and quantification of brain tissue loss in CrT^+/y^, CrT^+/–^, and CrT^–/y^ mice 7 days after neonatal HI (*n* = 5–11 for each group, as indicated). (**C**) Quantification of TUNEL^+^ cell death in the ipsilateral cerebral cortex and hippocampus in CrT^+/y^ versus CrT^–/y^ mouse brains 24 hours after neonatal HI (*n* = 3 for each). (**D** and **E**) Immunoblotting and quantification of the p-AMPK/autophagy and mTOR signaling pathway activity in the contralateral or ipsilateral (Con or Ipsi) hemisphere of P10 CrT^+/y^ versus CrT^–/y^ neonates 24 hours after unilateral HI. The violin plots in **E** are representative of 3 independent experiments, with *n* = 3 biological replicates; all other data are shown as mean ± SEM. Statistical significance was determined using 1-way ANOVA with Tukey’s multiple comparison post hoc test (**B**), Student’s *t* test (**C**), or 2-way ANOVA followed by Tukey’s multiple comparison post hoc test (**E**). (**F**) Anti-LC3B staining of HI-injured CrT^+/y^ versus CrT^–/y^ mouse brains at 24 hours of recovery (*n* = 5 for each). Scale bar: 20 μm.

Next, we tested the consequences of CrT deficiency on brain damage caused by neonatal HI insult. We found that 10-day-old CrT^–/y^ neonates (*n* = 9) had significantly greater brain atrophy than CrT^+/y^ (*n* = 11) or CrT^+/–^ (*n* = 5) mice 7 days after HI (Figure 5, A and B). At 24 hours after HI, the CrT^–/y^ neonates also showed more TUNEL^+^ cell death in the cerebral cortex and hippocampus than the CrT^+/y^ siblings (Figure 5C; *n* = 3 for each genotype). Similar to the aberrations in neonatal starvation and in vitro OGD, CrT^–/y^ neonates showed a greater increase in p-AMPKα and reduction in p62/SQSTM1 and p-ULK1 (Ser757) in the contralateral hemisphere 24 hours after HI, when compared with HI-injured CrT^+/y^ mice (Figure 5, D and E; *n* = 3 for each genotype). These differences were preserved in the ipsilateral hemisphere of HI-injured CrT^–/y^ mice, plus greater p-ULK1 (Ser 317) and further reduction in p-mTOR (Ser2448), p-S6, p-4E-BP, and p-ULK1 (Ser757), compared with HI-injured CrT^+/y^ neonates (Figure 5, D and E). Furthermore, CrT^–/y^ neonates showed greater anti-LC3B immunoreactivity compared with CrT^+/y^ mice in the ipsilateral hemisphere 24 hours after HI (Figure 5F; *n* = 5 for each genotype). Together, these results suggest a pivot toward p-AMPK/autophagy signaling away from the mTOR pathway in CrT^–/y^ neurons and neonatal brains with greater sensitivity to the OGD and HI insult, respectively.

### CrT deficiency causes greater infarction and ATP depletion after cerebral ischemia.

Intracerebroventricular injection of Cr diminished ischemic brain infarction in a previous report (12). Thus, we sought to test whether Cr/CrT deficiency inversely enlarges infarction or alters the signaling responses to cerebral ischemia. We used 16-day-old CrT^–/y^ mice for this study to avoid the confounding factor of their growth retardation in adulthood, as reported previously (19).

**Figure 6:**
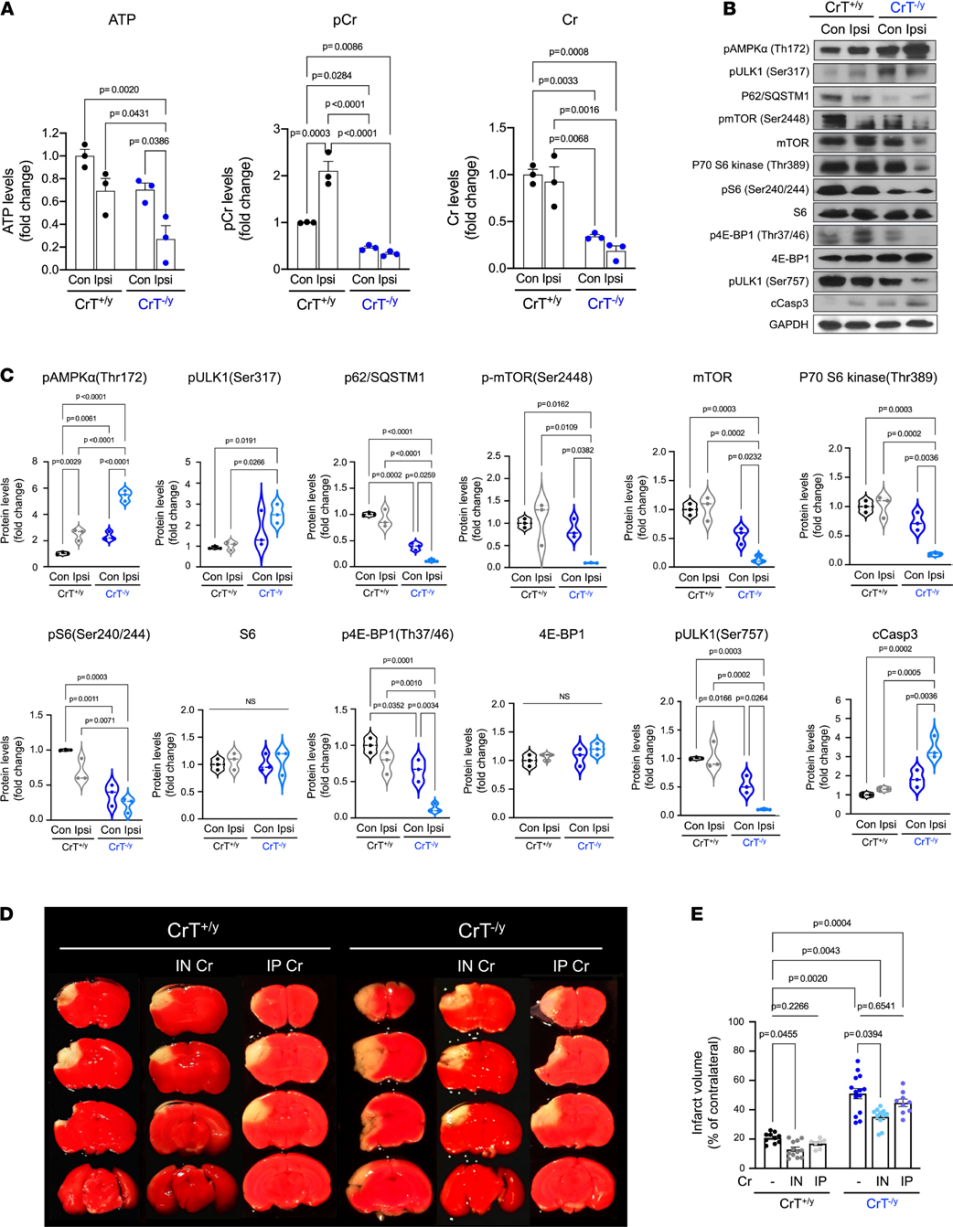
CrT deficiency causes greater infarct and ATP depletion after cerebral ischemia. (**A**) LC-MS quantification of the brain ATP, Cr, and PCr levels in contralateral (Con) and ipsilateral (Ipsi) hemispheres in CrT^+/y^ versus CrT^–/y^ mice 24 hours after photoactivation (*n* = 4 for each). (**B** and **C**) Immunoblotting and quantification of the p-AMPK/autophagy and mTOR signaling pathway activity in P16 CrT^+/y^ versus CrT^–/y^ mice 24 hours after photoactivation directed at the proximal branch of the middle cerebral artery. The protein expression levels in the contralateral hemisphere of CrT^+/y^ mice were used as the baseline. (**D** and **E**) Representative brain sections and quantification of the infarct size in untreated CrT^+/y^ (*n* = 10) and CrT^–/y^ mice (*n* = 14), intranasal 184 mg/kg Cr–treated CrT^+/y^ (*n* = 12) and CrT^–/y^ mice (*n* = 11), and intraperitoneal 200 mg/kg Cr–treated CrT^+/y^ (*n* = 7) and CrT^–/y^ mice at 16 days of age (*n* = 9). The intranasal and intraperitoneal Cr treatment was administered within 30 minutes after photoactivation. The violin plots in **C** are representative of 3 independent experiments, with *n* = 3 biological replicates; all other data are shown as mean ± SEM. All statistical analyses were performed using 2-way ANOVA followed by Tukey’s multiple comparison post hoc test. IN, intranasal; IP, intraperitoneal.

We induced photothrombosis at the proximal branch of the middle cerebral artery (MCA) in CrT^+/y^ and CrT^–/y^ mice and compared brain ATP, Cr, and PCr levels plus signaling responses in both hemispheres 24 hours after photoactivation (Figure 6, A–C; *n* = 3 for each condition). Similar to the trend in neonatal starvation, P16 CrT^–/y^ mice showed a marked reduction in brain ATP in the ipsilateral cortex after photothrombosis, when compared with the contralateral CrT^–/y^ and ipsilateral CrT^+/y^ cortex (Figure 6A). Similarly, immunoblotting showed a marked increase in p-AMPKα, p-ULK1 (Ser 317), and active caspase-3 plus a greater reduction in p62/SQSTM1, p-mTOR, mTOR, p-S6, p-4E-BP, and p-ULK1 (Ser757) in the ipsilateral cerebral cortex of CrT^–/y^ mice 24 hours after stroke (Figure 6, B and C). In addition, the infarct size was markedly increased in CrT^–/y^ mice (*n* = 14) when compared with the CrT^+/y^ siblings (Figure 6, D and E; *n* = 10). These results are very similar to those in neonatal HI, suggesting that CrT deficiency impairs brain energetics and the stress-adaptation responses to a wide range of neural insults.

### Intranasal delivery of Cr elevates brain Cr levels and reduces infarction in CrT^–/y^ mice.

Next, we tested whether i.n. application of Cr after stroke is an effective and better strategy for brain protection than i.p. injection of Cr. A recent study showed the benefits of i.n. application of Cr analog–loaded nanoparticles in CrT^–/y^ mice (29). We hypothesize that i.n. delivery of Cr also bypasses the BBB to protect against ischemia in CrT^–/y^ and wild-type mice, while i.p. injection of Cr is less effective due to a slow blood-to-brain transport (30, 31).

**Figure 7:**
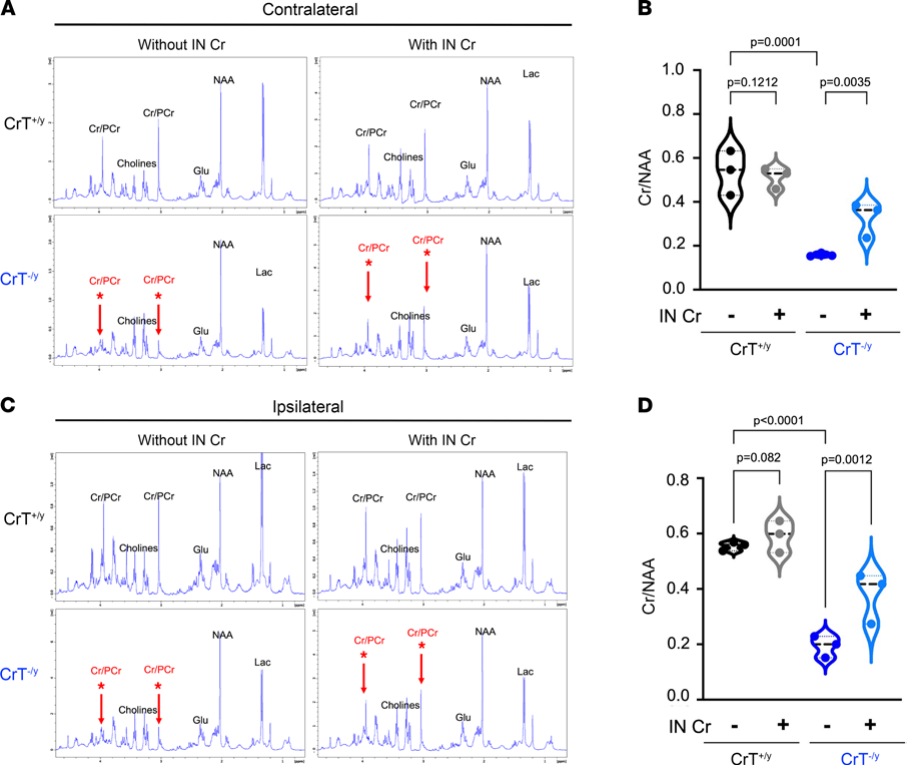
Proton–HR-MAS NMR showed elevation of brain Cr/PCr peaks by intranasal Cr treatment at cerebral ischemia. Proton–HR-MAS NMR analysis was performed to compare the contralateral (**A** and **B**) and ipsilateral (**C** and **D**) cerebral cortices of CrT^+/y^ and CrT^–/y^ mice 24 hours after photothrombosis. This analysis showed that intranasal Cr treatment elevated the Cr/PCr peaks and increased the Cr/NAA ratio in both contralateral and ipsilateral cortices of CrT^–/y^ mice, while its effects on CrT^+/y^ were minimal. The violin plots are representative of 3 independent experiments, with *n* = 3 biological replicates. All statistical analyses were performed using 2-way ANOVA followed by Tukey’s multiple comparison post hoc test.

Indeed, we found that i.n. application of 184 mg/kg Cr immediately after photothrombosis produced a significant reduction in infarct in CrT^–/y^ mice and a trend toward smaller infarct size in CrT^+/y^ mice, whereas i.p. injection of 200 mg/kg Cr was ineffective in either CrT^–/y^ or CrT^+/y^ mice (Figure 6, D and E). We then used proton–HR-MAS NMR to compare the effects of i.n. Cr supplementation in CrT^–/y^ or CrT^+/y^ mouse brains (Figure 7). This analysis showed that i.n. delivery of Cr elevated the Cr/PCr peaks and the Cr/*N*-acetylaspartate (Cr/NAA) ratio in the contralateral (Figure 7, A and B) as well as ipsilateral cerebral cortex of CrT^–/y^ mice (Figure 7, C and D). In contrast, i.n. delivery of Cr did not have obvious effects on the Cr/NAA ratio in either the contralateral or ipsilateral cerebral cortex of CrT^+/y^ mice (Figure 7, A–D), presumably due to the saturation by endogenous Cr.

**Figure 8:**
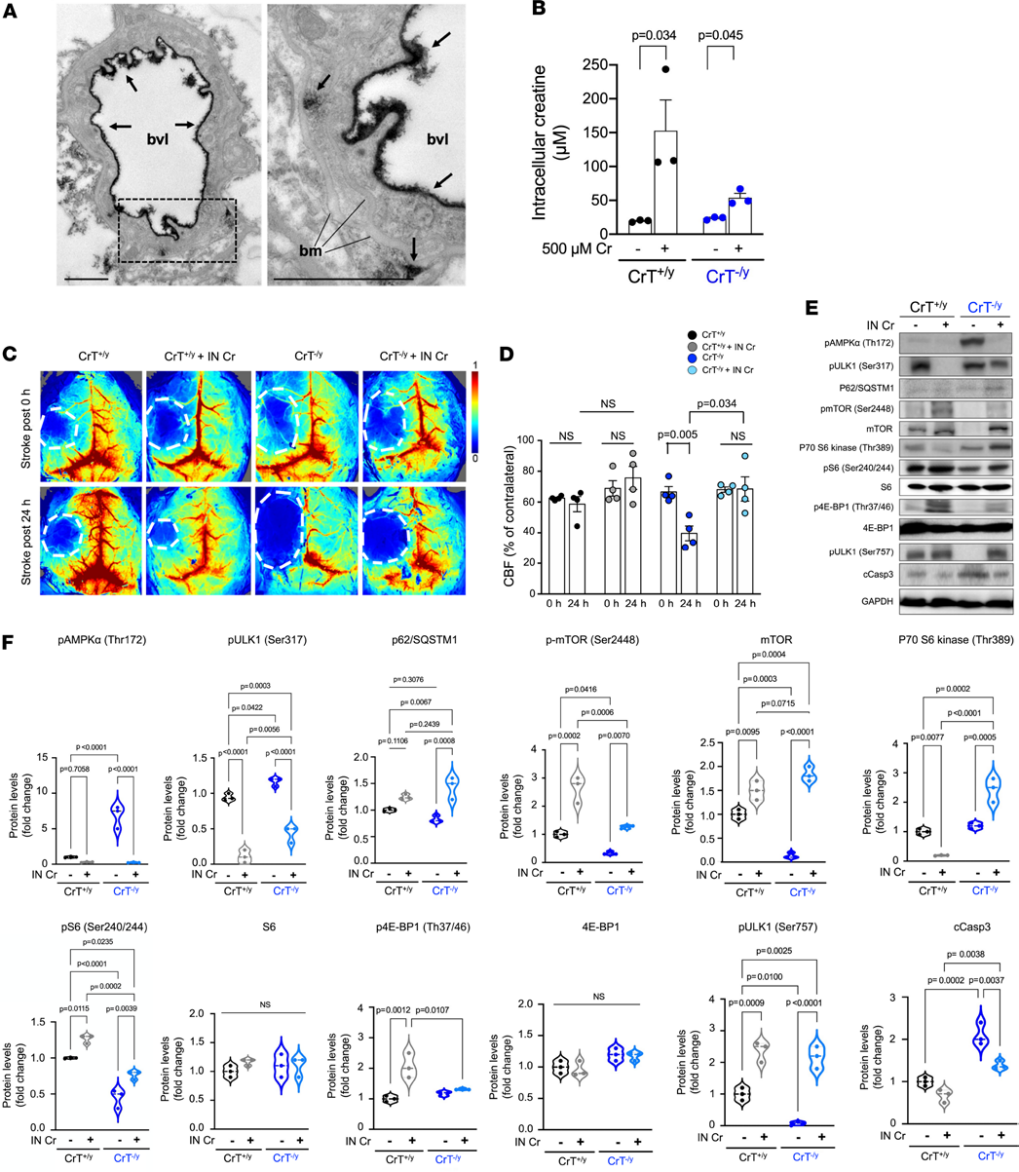
Post-stroke intranasal Cr delivery reduces brain injury and signaling imbalance. (**A**) CrT distribution in murine brain based on anti-CrT/anti–β-galactosidase immuno-EM labeling in CrT^+/–^ mice. Arrows indicate robust immunoreaction deposits on the extracellular surface of endothelial cells in the blood vessel lumen (bvl) and endothelial basement membrane (bm). Scale bars: 1 μm. (**B**) CrT^–/y^ neurons internalized Cr from the medium containing 500 μM Cr at approximately 40% efficiency compared with CrT^+/y^ neurons (*n* = 3). (**C** and **D**) Cerebral blood flow (CBF) was evaluated by laser speckle contrast imaging (LSCI) immediately after photothrombosis and reassessed 24 hours later in CrT^+/y^ and CrT^–/y^ mice, with or without intranasal Cr supplementation (*n* = 4 for each condition). (**E** and **F**) Immunoblotting and quantification of the p-AMPK/autophagy and mTOR signaling pathway activity in stroke-injured CrT^+/y^ and CrT^–/y^ hemispheres, with or without intranasal Cr supplementation, at 24 hours of recovery. Intranasal Cr supplementation showed significant attenuation of stroke-induced p-AMPK/autophagy signaling and better-preserved mTOR activity. The violin plots in **F** are from 3 independent experiments; all other data are shown as mean ± SEM. Statistical significance was determined using Student’s *t* test (**B**) or 2-way ANOVA followed by Tukey’s multiple comparison post hoc test (**D** and **F**).

In view of these results, we sought to confirm a report of CrT expression near the BBB (18). We initially tested 3 commercial anti-CrT antibodies, but all had strong cross-reactivity with CrT^–/y^ tissues. Therefore, immuno-EM staining against β-galactosidase (encoded by the *lacZ* gene) was used as a surrogate marker in CrT^+/–^ mouse brain, based on the knockout-first allele in our gene-targeting strategy (Figure 1A). This analysis showed intense extracellular immunoreaction deposits on the blood vessel lumen (arrows in Figure 8A), suggesting that CrT is expressed by the cerebral vascular endothelial cells. Of note, the anti–β-galactosidase immuno-EM analysis in CrT^+/–^mice cannot ascertain the subcellular distribution of endogenous CrT. RT-qPCR analysis showed a significantly reduced but residual amount of CrT (*Slc6a8*) mRNA in the brains of Tie2-Cre^Tg^; CrT^fl/y^ mice, suggesting the expression of CrT in cell types other than those in vascular endothelium in murine brains (Supplemental Figure 3; *n* = 3 for each genotype). Thus, we used gas chromatography–mass spectrometry (GC-MS) to compare the uptake of Cr by CrT^+/y^ and CrT^–/y^ cortical neurons from the medium. This analysis showed approximately 40% residual Cr uptake in CrT^–/y^ neurons compared with CrT^+/y^ neurons (Figure 8B; *n* = 3 for each condition), similar to the findings in CrT-null fibroblasts from patients (16). Together, these results suggest that i.n. delivery of Cr may bypass the BBB where CrT resides to assist Cr uptake, and that CrT^–/y^ neurons retain a reduced capacity for importing extracellular Cr.

Next, we assessed the effects of i.n. Cr delivery on post-stroke cerebral blood flow (CBF). We used laser speckle contrast imaging to compare the CBF immediately after photoactivation and after 24 hours of recovery in the same mouse, with or without i.n. Cr treatment. This analysis showed that i.n. delivery of Cr attenuated the deterioration of CBF 24 hours after photothrombosis in CrT^–/y^ mice (Figure 8, C and D; *n* = 4 for each group). Our results also suggest that i.n. Cr treatment improved CBF in the penumbra area in CrT^+/y^ mice 24 hours after photothrombosis (Figure 8, C and D).

Finally, we used immunoblotting to test the effects of i.n. Cr supplementation on the signaling responses in CrT^+/y^ and CrT^–/y^ mice 24 hours after photoactivation. In CrT^+/y^ mice, i.n. Cr treatment resulted in a marked reduction in p-ULK1 (Ser317) and an increase in p-mTOR and p-4E-BP (Figure 8, E and F; *n* = 3 for each condition). These signaling alterations plus a striking reduction in p-AMPKα, active caspase-3, and an increase in p62/SQSTM1, p-mTOR, mTOR, p-S6, and p-ULK1 (Ser757) was also found in CrT^–/y^ mice with i.n. Cr delivery 24 hours after photoactivation (Figure 8, E and F; *n* = 3 for each condition). Collectively, these results suggest that i.n. Cr supplementation partially averted the stroke-induced imbalance between autophagy and mTOR signaling in both wild-type and CrT^–/y^ mice.

## Discussion

CrT deficiency is the number one cause of cerebral Cr deficiency syndrome and leads to intellectual disabilities in approximately 2% of males (15, 16). Unlike those with Cr-synthesis-enzyme mutations, patients with CrT deficiency showed poor responses to oral Cr supplementation (10, 11). These clinical features underscore an unmet medical need and important functions of CrT in transporting Cr across the BBB. An effective strategy to supplement the brain Cr/PCr may benefit patients with congenital CrT deficiency, neurodegenerative disorders, and acute brain injury (4–6). Transgenic CrT-null mice are a valuable tool to address these issues, since the Cr biosynthesis pathway, the X-chromosome location of the CrT gene (*Slc6a8*), and the consequence of cognitive impairment due to CrT deficiency are conserved between humans and mice (1, 2). Herein, we discuss our findings of CrT-null mice in the context of (a) the roles of Cr and CrT in stress adaptation and brain energetics homeostasis and (b) potential treatment of CrT deficiency.

### Roles of Cr and CrT in brain energetics homeostasis and stress adaptation.

Cellular energetics is maintained by glycolysis, mitochondrial oxidative phosphorylation (OXPHOS), ATP regeneration by PCr through the Lohmann reaction (ADP + PCr → ATP + Cr), and a fine balance between autophagy (catabolism) and mTOR-mediated anabolism (1, 32). In skeletal muscles, Cr/PCr functions as an energy shuttle between the mitochondria where ATP is produced and the cytosol, where ATP is utilized but quickly regenerated by Cr/PCr (1–3). This elaborate scheme of energy shuttling and Cr/PCr-mediated ATP regeneration confers several advantages for muscle cells. First, PCr regenerates ATP at a rate 10 times faster than glycolysis and 40 times faster than OXPHOS, enabling muscle cells to cope with a sudden energy demand. Second, 1 proton is released from every ATP that is hydrolyzed to ADP, which would have lowered the cellular pH, but the proton is promptly recycled by Cr/PCr to regenerate ATP (33). Third, due to the smaller size of Cr, skeletal muscle cells store up to 10-fold more Cr than ATP as the energy reserve (1). Last but not least, Cr stimulates OXPHOS when it reenters the mitochondria through voltage-dependent anion channels, thus matching mitochondrial respiration with the cellular demand of ATP (34). Although Cr/PCr may have similar functions in the brain, the effects of CrT deficiency on stress adaptation and energy homeostasis remain uncertain.

**Figure 9:**
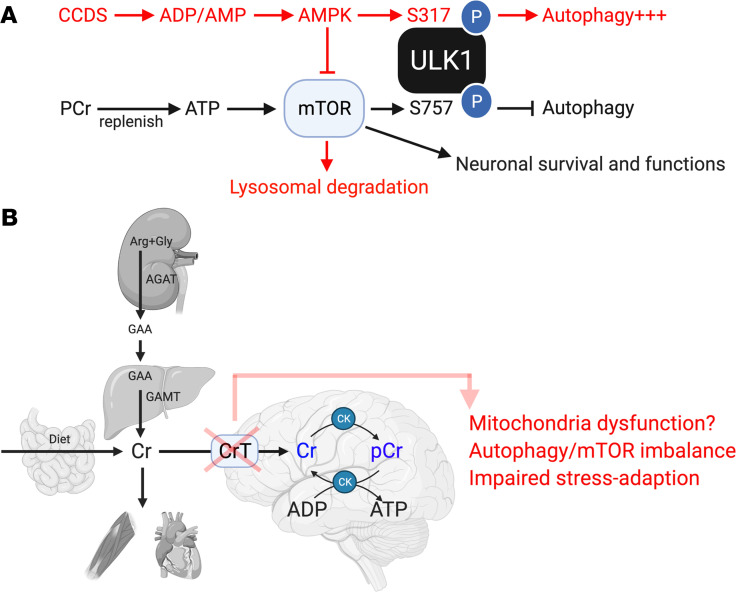
Potential functions of Cr and CrT in brain energetics and signaling homeostasis. Schematic depictions of (**A**) the PCr-mediated ATP regeneration and the default mTOR signaling responses under normal conditions (in the black text and arrows, respectively) and (**B**) the Cr biosynthesis pathway and the consequences of CrT deficiency (in red text and arrow).

Previous studies have shown that AGAT deficiency (and the ensuing Cr/PCr reduction) induces AMPK activation and a moderate ATP reduction in skeletal muscles (35, 36). We have found similar effects on ATP and p-AMPK, accompanied by a higher p-ULK1 (Ser317, proautophagy) to p-ULK1 (Ser757, antiautophagy) ratio and reduced mTOR phosphorylation, in 3- and 16-day-old CrT^–/y^ mouse brains without external stress. These results suggest a pivot toward autophagy (catabolism) from the mTOR (anabolism) signaling pathway to meet the basal energetics needs in CrT-deficient brains (Figure 9A). When an external stress (neonatal starvation, HI, or photothrombosis) was imposed, the CrT^–/y^ mouse brains showed a greater reduction in ATP and larger imbalance between autophagy and mTOR signaling than CrT^+/y^ mice. Moreover, the addition of the autophagy inhibitor 3-MA reduced the viability of CrT^–/y^ neurons after in vitro HI, suggesting that autophagy induction under HI is an adaptive response. These results suggest that Cr/PCr deficiency impairs brain energy homeostasis and stress-adaptation capacity, since the baseline autophagy is already elevated in the CrT^–/y^ mouse brains. Moreover, it has been shown that Cr depletion reduces OXPHOS complex III and IV activity and may promote myopathy-like mitochondrial pathology (36, 37). These results suggest that CrT deficiency may impair brain mitochondrial respiration and reduce stress-adaptation capacity (Figure 9B).

Interestingly, i.n. application of Cr after cerebral ischemia averted the worsening imbalance between autophagy and mTOR signaling and reduced the infarct size in both wild-type and CrT-null mice. These effects of Cr supplementation are even more striking when one considers that Cr lacks a high-energy phosphate group and cannot regenerate ATP directly. Although the exact mechanisms of Cr-mediated neuroprotection are uncertain, we suggest that Cr may directly stimulate mitochondrial respiration to increase ATP output, similar to a previous report (34), to preserve brain energetics homeostasis under crisis.

### Potential treatment of CrT deficiency.

Previous studies have shown cognitive deficits in multiple lines of CrT-deficient mice, but the neurocytological basis of these cognition deficits is poorly understood. In the present study, we showed that CrT-null mice carry a significant reduction in dendritic spines and synapses. These results support the suggestion that dysgenesis of dendritic spines and synapses is a pathological mechanism of intellectual disabilities (25). Moreover, since the mTOR and autophagy signaling pathways have opposing functions in dendritic spine formation (38, 39), the spine and synaptic aberrations in CrT-null mice may derive from a chronic imbalance between AMPK/autophagy and mTOR signaling pathways. For future research, it will be interesting to examine the ability of brain Cr supplementation to reverse the dendritic spine and synapse dysgenesis.

CrT-deficient patients show very poor responses to oral Cr supplementation (11), likely because plasma Cr cannot cross the BBB efficiently without CrT. Hence, there have been 2 strategies to overcome this therapeutic obstacle. One is oral supplementation of cyclocreatine (CCr), which crosses the BBB without CrT and can be phosphorylated by Cr kinases (20). Yet, phosphorylated CCr is 50- to 100-fold less efficient than PCr in regenerating ATP by Cr kinase (40). Moreover, the Lumos Pharma biopharmaceutical company has terminated its LUM-001 (CCr) program for treating CrT deficiency due to adverse effects found in animal studies. The other strategy is i.n. application of microemulsions loaded with the Cr derivative dodecyl Cr ester (DCE) to bypass the BBB and overcome the instability of DCE in blood (29). Other potential therapeutics include di-acetyl creatine ethyl ester (DAC), a novel Cr derivative, or 4-phenylbutyrate, a chemical that corrects some misfolded CrT mutant variants (41, 42).

In this context, our study shows that i.n. application of Cr reduces infarction and attenuates the signaling imbalance in both wild-type and CrT-null mice. Thus, i.n. delivery of Cr itself may be sufficient to correct CrT deficiency and harness the neuroprotective effects of Cr against acute brain injury. Future studies are warranted to examine the effects of i.n. Cr delivery against other brain injury models and test whether this treatment improves the cognitive functions and corrects the dendritic spine and synaptic defects in CrT-null mice. Finally, it is important to test whether there is an age window for cognitive improvement by i.n. Cr treatment in CrT-null mice. Outcomes of these studies will help to translate this therapeutic strategy into clinical practice.

## Methods

### Generation of CrT^–/y^ mice and genotyping.

We used an *Slc6a8*-targeted ES line [CSD24513, tm1a(KOMP)Wtsi; knockout-first, promoter-driven] in the KOMP Repository to generate CrT/*Slc6a8^–/y^* mice (24). The CrT knockout-first allele, including the *lacZ* expression reporter cassette, was produced by introducing loxP sites flanking exons 5–7 of the gene in ES cells by homologous recombination, as illustrated in Figure 1A. The CrT^+/–^ founder mice were produced in a mixed C57/sv129 background, and backcrossed with wild-type (CrT^+/y^) C57BL/6J mice for more than 6 generations. Germline transmission of the CrT-null allele was confirmed by PCR. All animal handling and maintenance were performed according to the regulations of the Institutional Animal Care and Use Committee at Emory University and the University of Virginia School of Medicine. The following primer sequences were used for PCR genotyping: F1: 5′-ATCCTCTGCATGGTCAGGTC-3′; R1: 5′-CGTGGCCTGATTCATTCC-3′; F2: 5′-TTGTAGGTGTGGAGGGCTTC-3′; R2: 5′-ACACACTCCCAAAAGGCTTG-3′; F3: 5′-GTGGCCACACCTGGAACACTCCTGACTGTGT-3′; R3: 5′-CCTTCCACACACAGAAGTAGACCAGCACCCA-3′; F4: 5′-ATCGTGTACTTCACTGCTACATTCCCCTACGTG-3′; R4: 5′-ATATGCACACCCTGCTCTGTGGCCATGAAG-3′; F5: 5′-CCTTCACAGCAGGGTCCTTAAATGC-3′; R5: 5′-GGAAGAAACAGGGCCATAGCATTCC-3′; F6: 5′-GGGATCTCATGCTGGAGTTCTTCG-3′; R6: 5′-TCCAGATCATCCTGATCGAC-3′; F7: 5′-GAGATGGCGCAACGCAATTAATG-3′; R7: 5′-AGCAGCAGCATGAAGAAGAACAAGG-3′; F8: 5′-GGCCTGGTCTAGCCTTCATT-3′; R8: 5′-CTCTGCCATGGTTCCTTTTG-3′.

### Protein extraction and Western blotting.

Previously described procedures were followed for these experiments (43, 44). The antibodies used were rabbit anti-AGAT (orb247515, Biorbyt); mouse anti-GAMT (sc-398936, Santa Cruz Biotechnology); mouse anti–synaptotagmin 1/2 (105011, Synaptic Systems); rabbit anti–PSD-95 (AB9708, Millipore); rabbit anti-Homer1 (ab211415, Abcam); rabbit anti–p-AMPK (Thr172) (2531S, Cell Signaling Technology); rabbit anti–p-UL K (Ser317) (12753, Cell Signaling Technology); rabbit anti-p62 rabbit (8025S, Cell Signaling Technology); rabbit anti–p-mTOR (Ser2448) (2971S, Cell Signaling Technology); rabbit anti-mTOR (2972S, Cell Signaling Technology); rabbit anti–p70 S6 kinase (Thr389) (9205S, Cell Signaling Technology); rabbit anti–p-S6 (Ser240/244) (5364S, Cell Signaling Technology); rabbit anti-S6 (2217S, Cell Signaling Technology); rabbit anti–p-4E-BP1(Thr37/46) (2855S, Cell Signaling Technology); rabbit anti–4E-BP1 (9644S, Cell Signaling Technology); rabbit anti–p-ULK (Ser757) (6888S, Cell Signaling Technology); rabbit anti-GAPDH (5174S, Cell Signaling Technology); rabbit anti-LC3B (ab48394, Abcam); and rabbit anti–cleaved caspase-3 (Asp175) (9661S, Cell Signaling Technology).

### RNA extraction, reverse transcription, and real-time PCR.

Previously described procedures were followed for these experiments (44). The samples were analyzed in triplicate and normalized versus the expression level of the GAPDH. The following primers were used for qPCR reactions: F2: 5′-TTGTAGGTGTGGAGGGCTTC-3′; R2: 5′-ACACACTCCCAAAAGGCTTG-3′; GAPDH-Forward: 5′-AGGTCGGTGTGAACGGATTTG-3′; GAPDH-Reverse: 5′-TGTAGACCATGTAGTTGAGGTCA-3′.

### HR-MAS NMR study.

The ex vivo NMR experiments and analysis of mice brain tissues were performed as previously reported (45). Briefly, a 1.5 mm punch (~10 mg) was taken from snap-frozen brain tissue and loaded into a sample rotor (4 mm ZrO_2_, Bruker Instruments), with 4 μL of deuterium oxide containing 100 mM sodium trimethylsilylpropionate-d4 (Sigma-Aldrich) added to obtain a frequency-lock signal and serve as an internal standard for chemical shift and metabolite quantification. HR-MAS NMR experiments were then carried out on a Bruker AVANCE 400 WB NMR spectrometer with a dedicated 4 mm HR-MAS probe. Spinning rates of samples were set to 2500 kHz (± 2 Hz) at 4°C. A T2-weighted, water-suppressed Carr-Purcell-Meiboom-Gill pulse sequence was used to acquire the data. The ^1^H-NMR spectra were recorded using key parameters as follows: repetition time of 2.0 seconds, spectral width of 4.8 kHz, 32K data points, and 256 transients. The presence and concentrations of selected metabolites in brain tissue samples were determined based on their chemical shifts and the corresponding integrals.

### Mitochondrial assays.

Mitochondrial superoxide was detected using the fluorescent MitoSox Red probe (Invitrogen). Primary CrT^+/y^ or CrT^–/y^ cortical neurons were incubated in Hank’s balanced salt solution (HBSS) with 2 μM MitoSox Red for 15 minutes at 37°C in a 5% CO_2_ atmosphere, washed with PBS, and the fluorescence assessed by Leica TCS SP8. For mitochondria morphology analysis, neurons were incubated with MitoTracker Green (Thermo Fisher Scientific) at a final concentration of 100 nM for 15 minutes at 37°C in a 5% CO_2_ incubator, and followed by a PBS wash before the fluorescence was assessed.

### Immunocytochemistry for light and EM.

EM analysis and immunolabeling were performed as previously described (46). Specifically, adult β-galactosidase–expressing CrT^+/–^ mice (*n* = 3) and wild-type mice as negative control (*n* = 2) were perfused transcardially with 4% paraformaldehyde, 0.2% picric acid, and 0.2% glutaraldehyde in 0.1 M phosphate buffer. Brains of 3-day-old mice (*n* = 12) were removed from the skull and immersion-fixed with the same fixative overnight at 4°C. The brains were sectioned into 100-μm-thick slices with a vibratome. For immunolabeling, slices were incubated with mouse anti–β-galactosidase antibodies (Promega, Z3788 lot 3718; dilution 1:1000) overnight at room temperature, and then with biotinylated anti-mouse IgG and the Elite ABC kit (all from Vector Laboratories). Ni-intensified 3,3′-diaminobenzidine-4HCl as a chromogen was applied. The slices were postfixed with 1% OsO_4_, stained with 2% uranyl acetate during dehydration, and embedded in Durcupan (ACM; Fluka, Buchs) on a microscope slide and coverslipped. Brain tissues were analyzed and photographed with an Axioplan 2 microscope (Zeiss). For EM investigations, regions of interest were re-embedded into Durcupan blocks and cut by a Reichert ultramicrotome into 70-nm-thick sections. The ultrathin sections were then stained with lead citrate and evaluated at 80 kV in a JEOL 1010 transmission electron microscope equipped with a Multiscan 792 digital camera (Gatan). The specificity of the anti–β-galactosidase antibody was confirmed by parallel immunolabeling in wild-type mice of similar age.

### Cr uptake assay and GC-MS.

Primary cortical neurons were prepared from P0 CrT^+/y^ or CrT^–/y^ pups. At 6 DIV, Cr monohydrate (500 μM; Sigma-Aldrich) was dissolved in Neurobasal medium containing 10% FBS, 100 U/mL penicillin and streptomycin, N2 supplement, and B27 supplement. After 24 hours, cells were washed with HBSS twice, harvested by trypsinization, washed twice in HBSS, pelleted, and stored at –80°C before they were further processed for the quantification of Cr by GC-MS, using stable isotope–labeled Cr as an internal standard. The final washing solution of the cells was analyzed by GC-MS, as previously described (47).

### Imaging and quantification of dendritic spines, synaptic density, and cell numbers.

(i) For dendritic spines analysis, YFP-labeled layer V pyramidal neurons in the motor and somatosensory cortex were imaged in coronal sections by confocal microscopy using a Leica SP8 system. The confocal files were analyzed by Imaris software (version 7.6.5, 64 bit; Bitplane). At least 1000 spines from 3 to 4 mice were constructed for each group. (ii) For synaptic density analysis, we performed confocal analysis of anti-synaptotagmin/PSD-95 signals and colocalization to test whether CrT knockout is associated with a reduction in synapses. To standardize the imaging analysis, a *Z*-stack of optical sections from the hippocampal CA1 region was captured from each section, and then synapses were counted in each 10 μm^2^ square. The measurements were collected from 10 randomly selected squares in each division for statistical analysis. (iii) For quantification of cell numbers, cells were counted in each condition and measurements were collected from 5 randomly selected 300 μm^2^ squares. Analyses of immunocytochemical staining and cell quantification were performed using ImageJ (NIH).

### Immunohistochemistry.

All immunohistochemistry was performed on 20-μm-thick frozen sections using standard procedures. The following primary antibodies were used: mouse anti–synaptotagmin 1/2 (105011, Synaptic Systems); rabbit anti–PSD-95 (AB9708, Millipore); and rabbit anti-LC3B (ab48394, Abcam).

### LC-MS.

Brain hemisphere tissue from CrT^+/y^ or CrT^–/y^ mice was homogenized and washed with PBS and deproteinized with an addition of 1 mL 60% methanol, followed by incubation at –20°C for 30 minutes. 1-Benzyl-4-phenyl-1,2,3-triazole (ITSD; Sigma-Aldrich) was added to the extraction solvents, which were sonicated for 15 minutes in an ice bath. The extract was centrifuged at 12,500*g* and 4°C for 15 minutes, and the supernatant was collected and stored at –70°C until analysis. The LC-MS/MS system used consisted of a Sciex API 3200 triple quadrupole mass spectrometer coupled with an Agilent 1200 HPLC. All MS analyses were performed on the mass spectrometer with the electrospray ionization source in negative mode. Selective reaction monitoring (SRM) mode was used for quantitative analysis. The MS parameters for SRM analysis of each analyte quantified were optimized with 100% buffer system at 300 μL/min flow rate to the values as follows: ion spray voltage, –4500 V; capillary temperature, 450°C; both the ion source gas 1 and ion source gas 2 were at 40 psi. The column used for the HPLC separation was a Gemini NX-C18 (3 mm, 100 × 2 mm, 110 Å) from Phenomenex. The HPLC separation was conducted using 100% ammonium acetate buffer in water (2 mM, pH = 10) as the mobile phase in an isocratic elution at 300 μL/min flow rate for 6 minutes. The sample was mixed with mobile phase and centrifuged and 20 μL of sample was injected.

### Primary cell culture, OGD, and cell viability.

Mouse cortical neurons were prepared from P0 CrT^+/y^, CrT^+/–^, and CrT^–/y^ pups. The cortex was mechanically dissociated by passing through a blue P1000 pipet tip 20 times and then filtered through a 70 μm nylon mesh (BD Biosciences). At 7 DIV, OGD treatment in vitro was used to most closely resemble in vivo hypoxic conditions. Cells were washed twice and incubated in glucose-free DMEM (Invitrogen) under hypoxic conditions (1% O_2_/5% CO_2_/94% N_2_ at 37°C) for 4 or 8 hours. At the end of the OGD, neurons were incubated in their regular Neurobasal medium and reintroduced to the regular atmospheric oxygen level for an additional 20 or 16 hours (reoxygenation). In each experiment, cultures exposed to OGD were compared with normoxic controls supplied with regular Neurobasal medium containing glucose and maintained in standard incubation conditions (normoxia; 21% O_2_/5% CO_2_ at 37°C). Cell viability was performed using a CCK-8 assay (Dojindo Molecular Technologies). Briefly, the main reaction was achieved by mixing CCK-8 solution with the culture medium and incubating for 1 hour in normal conditions. The absorbance values were read using a Molecular Devices SpectraMax M3 microplate reader at 450 nm. For analysis of inhibition of autophagy with 3-MA, cortical neurons were treated with 5 mM 3-MA (Sigma-Aldrich) for 24 hours following OGD.

### Neonatal HI surgery.

The murine pups of indicated groups were challenged with the Rice-Vannucci model of HI (unilateral common carotid artery ligation followed by 60-minute exposure to 10% oxygen at 37°C) at 10 days of age. The experimental paradigm of HI and brain tissue loss quantification were performed as described previously (43, 44).

### TUNEL assay.

Brains from indicated littermates were fixed using 4% paraformaldehyde at 4°C overnight with gentle agitation, cryopreserved in 30 % sucrose, frozen, and finally stored at –20°C until TUNEL assay was performed. TUNEL staining was used to identify apoptotic cells under light microscopy by using a Click-iT TUNEL Alexa Fluor 594 Imaging Assay kit (Thermo Fisher Scientific) according to the manufacturer’s instructions.

### Photothrombotic MCAO and stroke volume.

Photothrombotic middle cerebral artery occlusion (MCAO) was induced as described previously (48). Briefly, P16 CrT^+/y^ and CrT^–/y^ mice were anesthetized with isoflurane and placed securely under a dissecting microscope. The proximal left MCA was exposed and directly irradiated with a green-light laser (5 mW, 543.5 nm; Melles Griot) for 15 minutes, and Rose Bengal (Thermo Fisher Scientific) 5 mg/mL in saline was injected via the retro-orbital vein (25 mg/kg). To assess the infarct size, brains were removed and cut into 1-mm-thick coronal sections and stained with 4% 2,3,5-triphenyltetrazolium chloride in PBS for 20 minutes at 37°C, and then fixed in 4% paraformaldehyde for 10 minutes.

### Laser speckle contrast imaging.

A 2-dimensional laser speckle contrast imaging system following the manufacturer’s instructions (MoorFLPI-2, Moor Instruments) was used to evaluate the CBF dynamics in CrT^+/y^ or CrT^–/y^ mice (48). Briefly, anesthetized mice were placed in the prone position with their skulls exposed but unopened. The CBF was measured in both cerebral hemispheres and recorded immediately after the photothrombotic MCAO surgery before and after intranasal Cr delivery. Also shown are CBF images at selected time points from the entire recording. CBF was analyzed by the MoorFLPI software and is shown as arbitrary units in a 16-color palette.

### Statistics.

Data are summarized as mean and standard error of the mean (SEM) or displayed as violin plots with curves encompassing the distribution of the data. The violin plots are closed curves representing data distribution and encapsulate the median, range, and interquartile range, with each symbol representing a biological replicate. Statistical tests were run independently in GraphPad Prism 8.0. In short, Student’s *t* test for independent samples with unequal variances was used to compare 2 groups, while 1- or 2-way ANOVA followed Tukey’s multiple comparison post hoc test was used to compare 3 or more groups where necessary. A *P* value of less than 0.05 was considered significant. The number of mice tested are indicated in each figure legend.

## Author contributions

HRC and CYK wrote this manuscript. CYK, TD, HRC, and DML provided conceptualization of this study. HRC, XZB, YMM, YL, SW, HHZ, ISK, EMF, PR, HM, and YYS performed experiments and data analysis.

## Supplementary Material

Supplemental data

## Version 1 07/29/2021

In-Press Preview

## Version 2 09/08/2021

Electronic publication
