# Supplementary material for: Creatine transporter deficiency impairs stress adaptation and brain energetics homeostasis
Source: JCI Insight. 2021 Sep 8;6(17):e140173. doi: 10.1172/jci.insight.140173 (PMC8492331; doi:10.1172/jci.insight.140173)
Supplement: Supplemental data [file jciinsight-6-140173-s110.pdf]

Fig. S1 Chen et al.

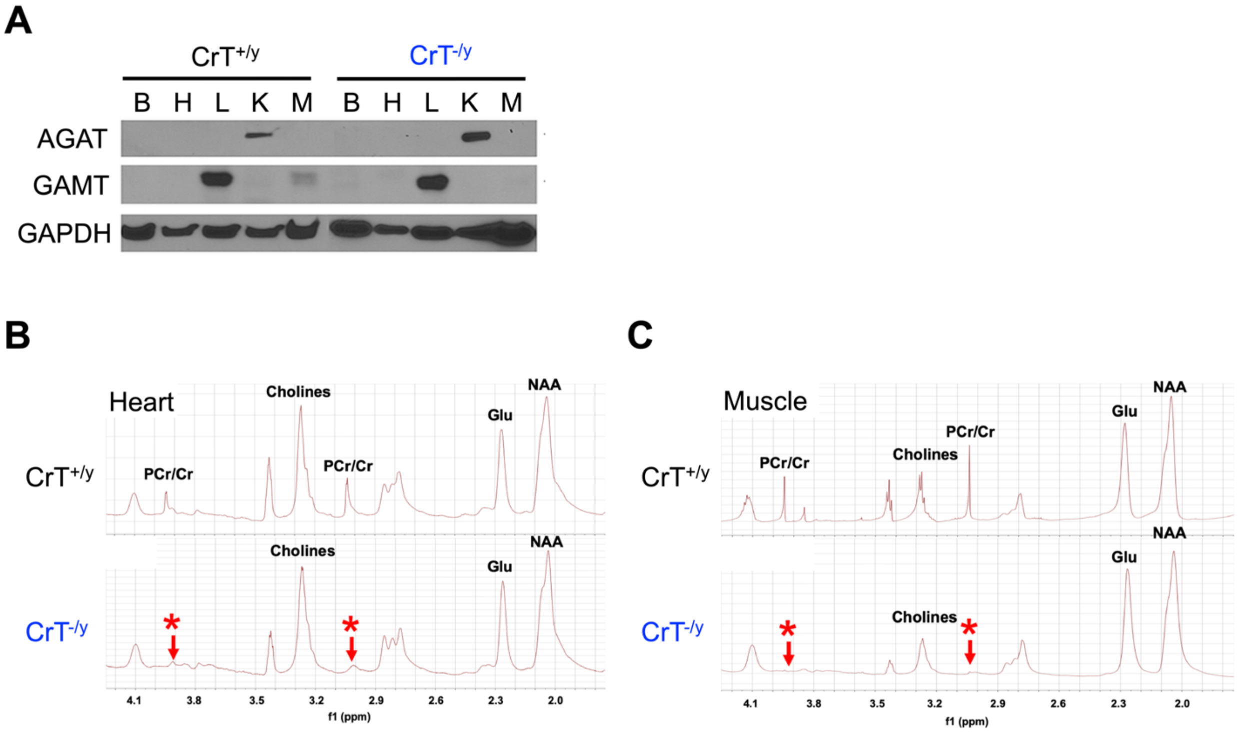

Supplementary Figure 1 ((A) Five-month-old CrT$^{-/y}$ mice retained the tissue-specific expression of AGAT (in the kidney, K) and GAMT (in the liver, L) without compensatory expression in the brain (B), heart (H), and skeletal muscles (M). (n=4 for each genotype). (B, C) Proton-NMR analysis of the heart and skeletal muscles in CrT$^{+/y}$ versus CrT$^{-/y}$ mice (n>5 for each genotype).

Fig. S2 Chen et al.

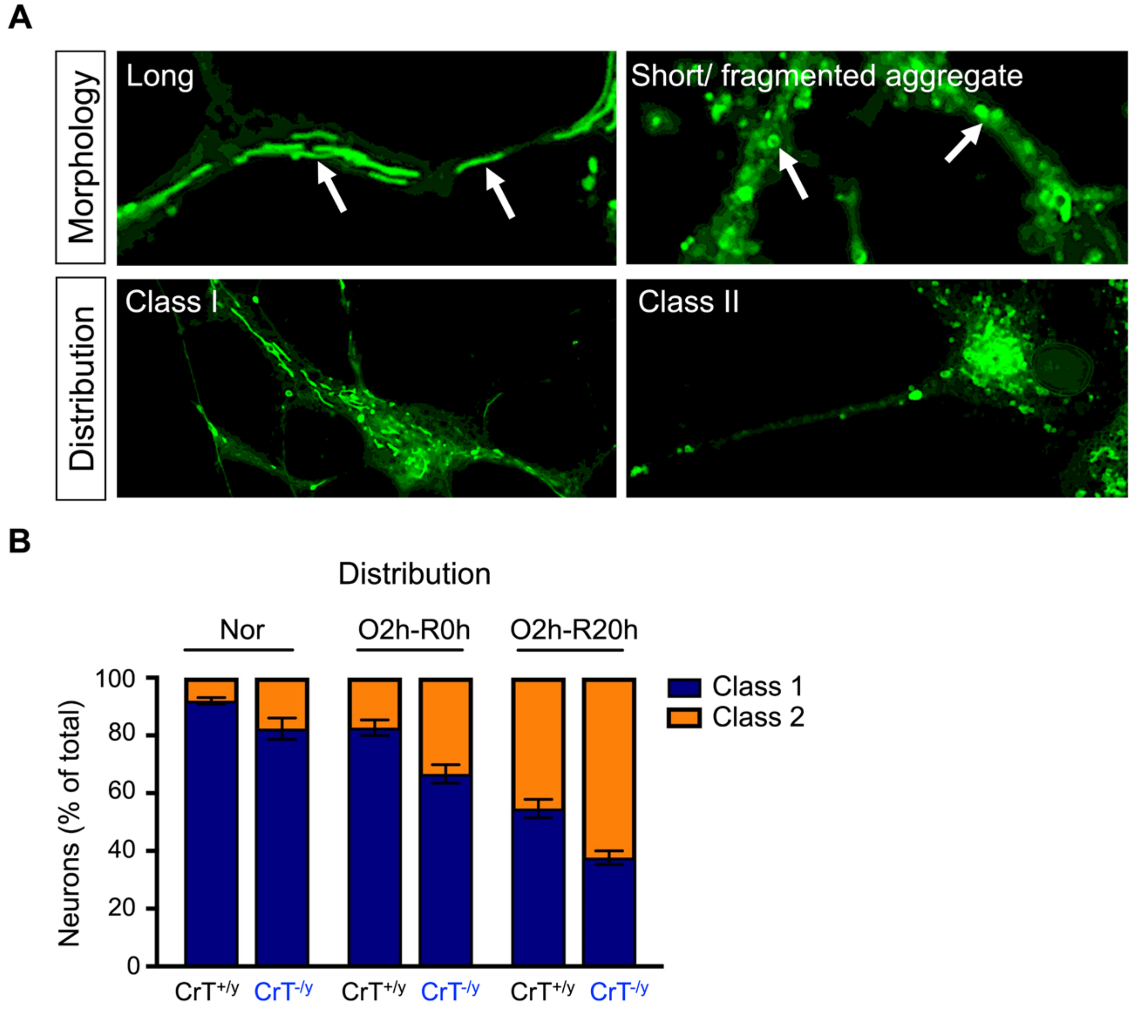

Supplementary Figure 2 Creatine transporter deficiency effects mitochondrial distribution in cortical neurons. (A) Representative confocal images of cortical neurons displaying the mitochondria morphology and distribution. (B) Histogram of the distribution of $CrT^{+/y}$ versus $CrT^{-/y}$ cortical neurons in the two class of distribution after 2h OGD challenged, followed by 0 or 20 h recovery (O2h-R0h and O2h-R20h), respectively. The mitochondrial morphology was visualized by MitoTracker Green. Shown are mean ± SEM; the *p*-value was determined by Student's *t*-test.

Fig. S3 Chen et al.

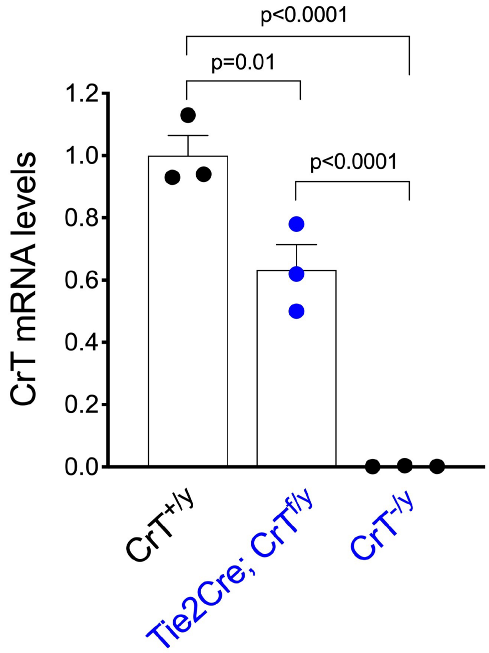

Supplementary Figure 3 CrT$^{f/f}$ and Tie2-Cre mice were crossed to produce the endothelium CrT-mutant mice (Tie2Cre^{tg}; CrT$^{f/y}$), which showed reduced expression of *CrT* mRNA in the brain (n=3 for each genotype), consistent with CrT expression on the vascular endothelium in mouse brain. Shown are mean ±SEM, all analyses were performed using one-way ANOVA followed by Tukey's multiple comparisons post-hoc test.
